# From Policy to Practice: The Achievements, Challenges, and Outlook of Birth Registration in Ghana

**DOI:** 10.1155/ijpe/8458061

**Published:** 2025-11-04

**Authors:** Sylvester Kyei-Gyamfi, Frank Kyei-Arthur, Stephen Afranie, Seth Bosompem Kissi, Amanda Kyei-Gyamfi

**Affiliations:** ^1^Department of Children, Ministry of Gender, Children and Social Protection, Accra, Greater Accra-Region, Ghana; ^2^Department of Environment and Public Health, University of Environment and Sustainable Development, Somanya, Eastern Region, Ghana; ^3^Centre for Social Policy Studies, University of Ghana, Legon-Accra, Greater Accra Region, Ghana; ^4^Births and Deaths Registry, Ministry of Local Government, Chieftaincy, and Religious Affairs, Accra, Ghana; ^5^Department of Chemical Engineering, Kwame Nkrumah University of Science and Technology, Kumasi, Ashanti Region, Ghana

**Keywords:** birth registration, Ghana, human rights, identity, policy implementation

## Abstract

Birth registration is a fundamental human right that serves as the first legal recognition of an individual's existence, yet global rates remain uneven, particularly in developing nations like Ghana. This paper employs a human rights-based approach (HRBA) to analyze Ghana's birth registration system, highlighting historical, administrative, and legal developments while assessing progress and persistent obstacles. Using a desk review methodology, the study synthesizes findings from government documents, scholarly articles, and reports from international organizations. It reveals that Ghana's birth registration framework, though improved through digitalization and integration with health services, still faces significant challenges including infrastructural disparities between urban and rural areas, cultural barriers, and gender biases. The study underscores the necessity for effective policy implementation that prioritizes inclusivity and addresses systemic barriers, framing birth registration not merely as an administrative task but as a critical component of governance and social equity. Ultimately, it questions whether birth registration in Ghana is a national priority or has become a neglected necessity.

## 1. Introduction

Birth registration is a fundamental human right and serves as the first legal recognition of a person's existence [1–3]. It establishes a child's identity, nationality, and access to social services, including education, healthcare, and social protection. Despite its significance, global birth registration rates remain uneven, with developed countries achieving near-universal coverage, while developing nations, particularly in Africa and Asia, continue to struggle with low registration rates [4]. These disparities are influenced by factors such as weak civil registration systems, sociocultural beliefs, financial constraints, and geographical barriers [5, 6]. In some cases, governments in low-income countries lack the resources and infrastructure to ensure birth registration reaches all communities, particularly those in remote areas [7, 8].

The importance of birth registration extends beyond legal identity. It is a crucial component in national planning and governance, as it enables governments to design and implement policies effectively. A comprehensive civil registration and vital statistics (CRVS) system aids in population data collection, social welfare planning, and resource allocation [9].

Globally, challenges persist in achieving universal birth registration. In many parts of Africa and Asia, factors such as cultural perceptions, illiteracy, and administrative inefficiencies hinder birth registration efforts [10–12]. In some communities, traditional naming ceremonies take precedence over official registration, causing delays or nonregistration of births [7, 8, 13]. The cost of registration required documentation, and accessibility to registration centers further complicate the process [5]. Some parents may be unaware of the importance of birth registration or lack the means to complete it within the legal timeframe [5, 7, 8].

In Ghana, birth registration has seen gradual progress over the years. The country introduced a formal registration system in the early twentieth century and has since undertaken various policy reforms to enhance coverage (Ministry of Gender, Children and Social Protection [MoGCSP], [1, 2, 14]. The Births and Deaths Registry (BDR), under the Ministry of Local Government, Chieftaincy, and Religious Affairs is responsible for overseeing the birth registration process [13]. Despite efforts to streamline the system, issues such as inefficiencies in data management, insufficient funding, and lack of coordination among stakeholders remain significant barriers [13]. There is also a persistent gap between urban and rural birth registration rates due to disparities in infrastructure and outreach programs [13].

Recognizing the need for improvement, Ghana has implemented various measures to increase birth registration rates. These include the digitalization of birth registration records, integration of birth registration with health services, and mobile registration units targeting rural and hard-to-reach communities. Public awareness campaigns have also been initiated to sensitize parents about the importance of timely birth registration [13]. While these interventions have yielded positive results, challenges such as delays in processing birth certificates, legal and procedural bottlenecks, and limited capacity of registration officials continue to hinder full-scale success [13].

This paper examines the status of birth registration in Ghana by analyzing the challenges that hinder universal registration, assessing the measures implemented to enhance coverage, and evaluating the achievements made over the years. The study further explores the policy and practical implications of birth registration for governance, social development, and children's rights protection. By critically examining the historical, administrative, and legal developments surrounding birth registration in Ghana, this paper is aimed at assessing the progress made as well as the persistent obstacles that hinder the system. Through this analysis, it seeks to answer the fundamental question: Is birth registration in Ghana a national priority or merely a neglected necessity? By evaluating the efficacy of existing policies, the accessibility of registration services, and the socioeconomic implications of unregistered births, the paper will provide insights into the status of birth registration as an essential element of identity and legal recognition for all citizens.

## 2. Theoretical Foundation: Human Rights-Based Approach (HRBA)

The HRBA provides a strong theoretical foundation for understanding birth registration as a critical component of human rights protection. The HRBA framework asserts that all individuals have inherent rights by virtue of being human, and states have obligations to ensure these rights are upheld [15]. Under this approach, birth registration is not merely an administrative process but a legal and moral obligation of the state to recognize and protect every child's identity and rights [1, 2].

The HRBA is rooted in international legal instruments, including the United Nations Convention on the Rights of the Child (UNCRC), which mandates birth registration for every child without discrimination [1, 2]. Article 7 of the CRC explicitly states that every child has the right to be registered immediately after birth and to acquire a nationality [16]. Failure to register births leads to a lack of legal recognition, making children invisible in the eyes of the state and vulnerable to rights violations [6]. Without official documentation, children may be denied access to education, healthcare, and social services, reinforcing cycles of poverty and marginalization [1, 2].

From a policy perspective, the HRBA calls for a shift from viewing birth registration as a privilege to recognizing it as an enforceable right. This requires governments to eliminate systemic barriers, ensure universal access to registration services, and prioritize the inclusion of marginalized and vulnerable populations [1, 2]. In Ghana, while legal frameworks exist to mandate birth registration, enforcement remains inconsistent [13]. Many rural and underserved communities continue to face challenges due to logistical constraints, financial barriers, and lack of awareness [13].

To systematically apply the HRBA framework, this study assesses Ghana's birth registration system through the five key HRBA principles: participation, accountability, nondiscrimination, empowerment, and legality.  Table 1 below presents Ghana's current status and identifies specific gaps.

Table 1 illustrates that although Ghana has made major improvements towards aligning its legal frameworks with international standards, considerable gaps persist in ensuring participation, accountability, and nondiscrimination. The systematic application of HRBA underscores the necessity for enhanced community engagement, improved accountability mechanisms, and targeted interventions for marginalized populations. Furthermore, the HRBA is in alignment with the sustainable development goals (SDGs), particularly Goal 16.9, which seeks to provide legal identity for all, including birth registration, by 2030. Ensuring universal birth registration is not only a human rights obligation but also a fundamental economic priority that is essential for governance, social equity, and national security [1, 2]. By integrating HRBA principles into Ghana's birth registration policies, the government has the opportunity to enhance compliance, increase accessibility, and fortify national identity systems.

## 3. Methods

This study employs a desk review approach, drawing on secondary sources to examine birth registration in Ghana. The data sources include government policy documents, statistical reports, scholarly journal articles, and publications from international organizations such as UNICEF, WHO, and UNHCR. National reports and academic studies provide insights into birth registration trends, challenges, and policy interventions.

A systematic and structured search strategy was employed to improve methodological transparency. Searches were performed from January to March 2024 utilizing academic databases (Google Scholar, JSTOR, PubMed, and Scopus), government archives (BDR and Births and Deaths Registry and institutional repositories (UNICEF, United Nations Development Program (UNDP), Plan International, and African Union). A review of gray literature, including unpublished reports and policy briefs, was conducted to ensure comprehensive coverage. The search strategy entailed the integration of keywords including “birth registration,” “civil registration,” “legal identity,” “child rights,” “Ghana,” “Sub-Saharan Africa,” “policy implementation”,“gender and birth registration,” and” culture and birth registration”. Sources published from 2010 to 2023 were prioritized to encompass recent developments and long-term policy trajectories. Studies were included based on the following criteria: (i) they addressed birth registration in Ghana or similar Sub-Saharan African contexts, (ii) they analyzed the legal, sociocultural, or institutional aspects of registration, and (iii) they were sourced from credible peer-reviewed journals, governmental bodies, or international organizations. Publications that concentrated solely on alternative identity systems without clear connections to birth registration were omitted.  Table 2 presents a summary of the databases searched and the key terms utilized.

Source selection was determined by relevance, credibility, and recency, prioritizing literature specific to Ghana while also incorporating comparative evidence from Africa and globally to contextualize the findings. This ensured that the analysis was based on contemporary policy discussions and historical viewpoints regarding the development of civil registration systems.

This review synthesizes diverse literature to provide an evidence-based analysis of the status of birth registration in Ghana, examining whether it is prioritized as a policy issue or remains overlooked. The findings enhance policy discourse by pinpointing implementation gaps and suggesting strategies to improve birth registration coverage and accessibility. Furthermore, effective practices from other nations are emphasized as possible frameworks for enhancing Ghana's birth registration system.

## 4. Birth Registration in Context

### 4.1. Definition and History

Birth registration is the official recording of a child's birth by a government authority, usually within a civil registration system [1, 2, 13]. It serves as the first legal recognition of an individual's existence and provides a gateway to accessing rights and services such as nationality, education, and healthcare [17]. Without birth registration, individuals may face difficulties in obtaining identity documents, securing employment, and participating in civic life. Historically, birth registration has varied across cultures and civilizations. Ancient Egypt, Greece, and Rome practiced forms of birth recording, mainly for taxation and military conscription purposes. Religious institutions, particularly in medieval Europe, played a significant role in birth documentation, maintaining parish records as the primary means of birth registration [18]. In Africa, traditional methods of birth acknowledgment existed, often through naming ceremonies or oral declarations. However, formal birth registration systems were largely introduced during the colonial era, influenced by European civil administration [17]. These colonial structures laid the foundation for modern civil registration systems in many African countries, including Ghana.

### 4.2. Types of Birth Registration Systems Around the World

Birth registration systems across the world vary significantly based on governance structures, administrative capabilities, and the level of integration with other national systems. Broadly, these systems can be categorized into centralized, decentralized, and hybrid models.

A centralized birth registration system is one in which a single national agency is responsible for recording all births within the country. This model ensures uniformity, standardization, and security of birth records, reducing inconsistencies that may arise from multiple points of data collection [1, 2]. Countries such as Sweden and Japan operate centralized registration systems, where all birth records are stored in a national database, ensuring easy retrieval and data accuracy [17]. The centralized approach enhances efficiency but requires significant government investment in digital infrastructure and personnel training.

In contrast, a decentralized birth registration system is managed by local or regional authorities, allowing for flexibility in record-keeping. This system is prevalent in large and highly populated countries such as India and Nigeria, where local governments are given the responsibility of birth registration [17]. While decentralized models facilitate accessibility for local populations, they often result in inconsistencies due to variations in administrative practices and resource availability. In some cases, decentralization has led to underreporting, particularly in rural areas where local authorities lack the necessary infrastructure to effectively register births [19].

A hybrid birth registration system combines elements of both centralized and decentralized models. Countries such as Ghana and South Africa have adopted this approach, where local offices collect birth data but report to a central authority for national consolidation [9]. This structure allows for greater accessibility while maintaining data integrity at the national level. Hybrid models often integrate birth registration with healthcare and social services, making it easier to capture birth records at hospitals and community health centers.

In many African countries, birth registration remains a challenge due to infrastructural deficits, financial constraints, and a lack of awareness. However, innovative solutions have been implemented to improve registration rates. For instance, Rwanda and Kenya have integrated birth registration with health services, allowing hospitals and clinics to serve as registration points [1, 2]. Additionally, mobile registration units have been deployed in remote areas to ensure wider coverage, particularly for marginalized communities.

### 4.3. Challenges of Birth Registration Around the World

Despite global efforts to ensure universal birth registration, significant challenges persist, particularly in low-income and rural communities. Several barriers hinder the effective implementation of birth registration systems, including geographical constraints, financial limitations, cultural practices, and administrative inefficiencies.

One of the most significant challenges is geographical barriers. In many remote and hard-to-reach areas, registration offices are located far from communities, making it difficult for families to complete the registration process [19]. Transportation difficulties, especially in regions with poor road networks, further exacerbate the problem. In response, some governments have introduced mobile registration units to bring birth registration services closer to underserved populations [1, 2].

Financial constraints also pose a significant obstacle to birth registration. While some countries offer free registration within a limited timeframe after birth, others impose fees that may be prohibitive for low-income families. In addition to registration fees, indirect costs such as transportation and documentation expenses deter many parents from registering their children's births [17]. This financial burden disproportionately affects families in rural areas and contributes to disparities in birth registration rates between urban and rural populations.

Cultural and religious practices influence birth registration rates in many parts of the world. In some communities, traditional naming ceremonies take precedence over legal registration, leading to delays or complete neglect of the process [20]. Certain indigenous groups and religious sects view birth registration as unnecessary or incompatible with their cultural beliefs, resulting in low compliance levels.

Additionally, legal and administrative bottlenecks impede birth registration efforts. Bureaucratic inefficiencies, outdated legislation, and a lack of coordination among relevant government agencies often slow down the process [17]. In many developing countries, manual record-keeping systems contribute to data inaccuracies and delays in issuing birth certificates. To address this issue, some nations have introduced digital birth registration systems to streamline processes and improve data management. Countries like Bhutan and Bangladesh have successfully implemented electronic registration systems, enhancing efficiency and accessibility (United Nations Children's Fund [UNCF]) [21].

Governments and international organizations have adopted various strategies to overcome these challenges. Health system integration has proven to be an effective approach in countries such as Ghana, Tanzania, and Uganda, where birth registration is linked with maternal and child healthcare services [1, 2]. This strategy ensures that newborns are registered at healthcare facilities shortly after birth, increasing coverage rates. Additionally, public awareness campaigns have been launched to educate parents about the importance of birth registration and the long-term benefits it provides to children and families [9].

### 4.4. Birth Registration System in Ghana

#### 4.4.1. A Historical and Conceptual Overview

**Figure 1:**
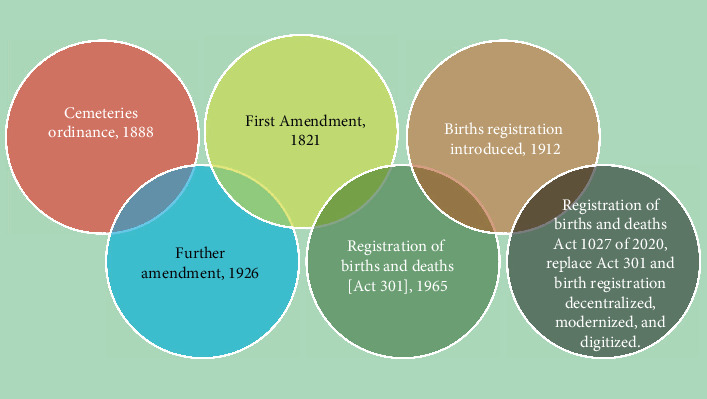
Historical and conceptual overview of birth registration in Ghana.

Birth registration in Ghana has undergone significant evolution over the past century, transitioning from an initial focus on death registration to a comprehensive system governed by modern legislation [13].  Figure 1 traces this progression, highlighting key legislative milestones.

The journey began in 1888 with the Cemeteries Ordinance, which prioritized death registration and led to many individuals being undocumented at birth. This ordinance was amended in 1891 but still lacked a formal birth registration system. A pivotal change occurred in 1912 with the introduction of the Registration of Births, Deaths, and Burials Ordinance, marking the official start of birth registration, though coverage was limited and not universal. An amendment in 1926 sought to strengthen this registration framework. In 1965, the Registration of Births and Deaths Act (Act 301) formally established the BDR under the Ministry of Local Government, aimed at improving record-keeping and increase coverage, particularly in rural areas. The latest legislative milestone, the Registration of Births and Deaths Act 1027 of 2020, replaced Act 301 and seeks to decentralize, modernize, and digitize the registration process with a focus on accessibility and accuracy.

Currently, the BDR plays a crucial role in Ghana's socioeconomic development, providing essential demographic data. Its vision is to achieve universal birth and death registration, ensuring that every child's right to identity and legal recognition is upheld.  Figure 1 captures these developments, demonstrating how birth registration has evolved from colonial-era ordinances into a structured, modernized system. Continuous policy improvements and technological integration are aimed at closing existing gaps in birth registration and fulfill the commitment to universal civil registration.

#### 4.4.2. Situational Analysis

Birth registration is crucial as it provides a child with their first legal recognition and establishes their legal identity for life, culminating in the issuance of a birth certificate. Without this document, children may be excluded from essential services such as education, healthcare, and other rights. In Ghana, four out of every 10 children are not registered at birth. Furthermore, even when children are registered, many lack proof of registration. Approximately 15% of registered children under the age of five do not possess a birth certificate, putting them at risk of exclusion from accessing basic services [22].

**Figure 2:**
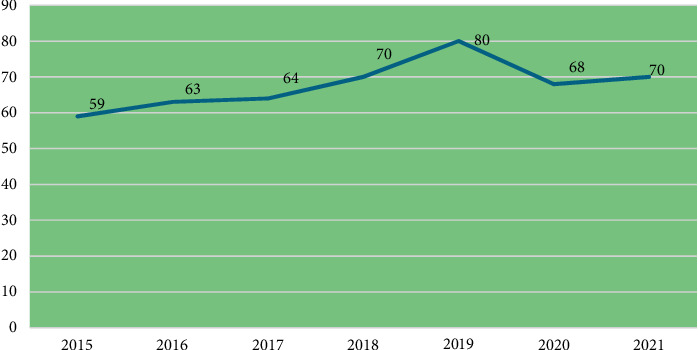
Trend in birth registration in Ghana (2015–2021).

Figure 2 shows the percentage of births registered from 2015 to 2021. From the figure, it would be observed that birth registration increased remarkably from 59% in 2015 to 80% in 2019 but fell to 70% in 2021. The multiple indicator cluster surveys (MICSs) in Ghana highlight several key factors that influence birth registration across different demographics, including sex, age, educational level, socioeconomic status, religion, place of residence, and wealth index [23]. Notably, there are significant disparities in registration rates between male and female children, with boys often being registered at higher rates due to cultural biases that prioritize male offspring. Families frequently focus on registering male births, leading to a discrepancy in official records for female children. Age is another critical factor, as younger children, particularly those born recently, tend to have lower registration rates. This trend is influenced by increasing awareness and improved access to registration services, resulting in better outcomes for newer generations as governmental and NGO initiatives ramp up.

Educational attainment affects birth registration, with parents possessing higher levels of education more likely to understand the importance of registration and the associated legal benefits. MICS data indicate that households led by individuals with secondary education or higher demonstrate significantly better registration rates compared to those with only primary education or none at all. This highlights the need for targeted outreach aimed at less educated communities. Socioeconomic status, measured through the wealth index, also correlates with registration rates. Families in higher wealth quartiles tend to register their children's births more frequently, driven by better access to resources, heightened legal awareness, and greater mobility to reach registration offices. In contrast, lower wealth quintiles face financial and logistical barriers that hinder their ability to complete the registration process.

### 4.5. Ghana's Commitment to Birth Registration

The Ghanaian government has increasingly recognized birth registration as a national priority due to its critical role in ensuring legal identity, access to social services, and national planning. This commitment aligns with SDG 16.9, which is aimed at providing legal identity for all by 2030. Recognizing the importance of birth registration, the government has taken several policy and administrative measures to strengthen the system. To enhance birth registration coverage, the Ghanaian government has implemented several key initiatives.

#### 4.5.1. Digitalization of Birth Records

The digitalization of birth records in Ghana is a significant initiative aimed at improving the efficiency and accuracy of the registration process [9]. Electronic registration systems have been introduced to streamline the collection and management of birth data, reducing reliance on manual record-keeping, which is often prone to errors. This transition to digital systems facilitates real-time data entry and retrieval, enhancing the overall reliability of birth statistics. Additionally, digitalization allows for better tracking of registered births, making it easier for authorities to identify unregistered children and implement targeted outreach programs [22].

A key component of this digitalization effort is the m-Birth Project, introduced in Ghana in December 2015 with a prepilot in Greater Accra and fully implemented from 2017 with support from UNICEF Ghana and the BDR. According to UNICEF Ghana [24], the initiative has significantly improved the efficiency of birth registration processes, particularly in underserved and remote areas, by leveraging mobile technology. More recent UNICEF data highlights that birth registration coverage in Ghana reached around 70% of children under five by 2021, with the m-Birth platform contributing to this progress [25]. The project seeks to facilitate timely and universal birth registration, ensuring that children receive legal identity through a birth certificate, which is crucial for accessing education, healthcare, and social protection services. This innovative initiative leverages mobile technology to enhance the birth registration process, particularly in rural and remote areas where access to traditional registration services is limited. The project is aimed at facilitating timely and efficient birth registration for children and ensuring that all children receive a legal identity through the issuance of a birth certificate, which is essential for accessing services such as education and healthcare.

The m-Birth Project has achieved notable success, significantly increasing the rate of birth registrations in targeted areas. By enabling registration officers to capture birth data electronically, the project simplifies the registration process and reduces delays. Community awareness campaigns conducted under this initiative have educated parents on the importance of birth registration and the services available through the m-Birth system. Furthermore, the project has fostered partnerships with local government bodies and community leaders, enhancing trust and cooperation within the registration process.

Despite these successes, the m-Birth Project faces challenges, including infrastructure limitations in terms of mobile and internet connectivity, which can hinder operation in some remote areas. Data security concerns regarding the protection of sensitive information collected during registration also pose challenges. Cultural beliefs in certain communities may discourage families from registering their children, and resource constraints can limit the project's expansion and sustainability. Overall, the digitalization of birth records, exemplified by initiatives like the m-Birth Project, is crucial for achieving universal birth registration in Ghana. These advancements not only improve the quality of data for planning and policymaking but also ensure that more children are recognized legally, thereby enhancing their access to essential services.

#### 4.5.2. Health System Integration

Health system integration in Ghana has been significantly enhanced to support the early registration of newborns (UNHCR, 2020). Maternity and postnatal care services are now linked with birth registration processes, ensuring that parents are informed about the importance of registering their children immediately after birth [26, 27]. According to the GSS [28] between 2016 and 2018, Ghana achieved approximately 70% birth registration coverage among children under five, with a gap observed between urban (around 80%) and rural areas (around 64%). These units are designed to bring registration services directly to communities that may lack easy access to fixed registration offices. By deploying trained personnel and necessary equipment to these areas, the government aims to increase birth registration coverage significantly. Mobile registration units not only facilitate the registration process but also serve as awareness campaign platforms to educate families about the importance of legal documentation for their children. This initiative has proven effective in reducing urban–rural disparities in registration, thereby contributing to the overall goal of universal birth registration in Ghana.

#### 4.5.4. Expanded Community Outreach Programs

The government has significantly expanded community outreach programs aimed at educating parents about the importance of birth registration, particularly in rural areas where awareness is often low. According to UNICEF Ghana [28], about 70% of Ghanaian children under five had their births registered by 2017/18, with a substantial urban–rural gap remaining (80% in urban areas vs. 64% in rural areas). These programs involve local health workers and community leaders who conduct workshops and informational sessions to inform families about the benefits of registering their children's births. By emphasizing the legal rights and access to services that come with birth registration, such as education and healthcare, these outreach efforts are aimed at changing cultural perceptions and encouraging parents to prioritize registration. This grassroots approach is crucial in areas where traditional practices may overshadow legal requirements, ensuring that more children receive official recognition at birth.

#### 4.5.5. Integration With National Identification Programs

The Government of Ghana has recently integrated birth registration with national identification programs, introducing the Ghana Card at Birth system to ensure newborns are linked to the National Identification Authority (NIA) database from the point of birth. Health facilities capture biometric and demographic details at birth via DHIS e-tracker and Lightwave, transmit to the BDR, and subsequently into the NIA system [29]. In addition, joint registration drives with the NIA, and the National Health Insurance Authority have been designed to enrol younger age groups (e.g. ages 6–14 years) under the Ghana Card program, with about 6.2 million children in this age group targeted for registration between October 2024 and March 2025 [29].

This integration is intended to streamline identification, reduce duplication, and improve the accuracy of civil records. It also ensures that children from birth are documented in the national identification database, strengthening planning, governance, and access to public services.

#### 4.5.6. Legislative Reforms

The government has introduced legal reforms to reduce financial barriers to birth registration, particularly for infants. Under the Registration of Births and Deaths Act, 2020 (Act 1027), birth registration within the first 12 months of a child's life is free of charge [30]. Following this policy environment, birth registration coverage in Ghana increased from about 63% in 2016 to approximately 80.4% in 2019 [31] reflecting broader improvements in compliance and outreach. These reforms are aimed at eliminating fees and other cost barriers so that low-income households are not discouraged from registering their children. Making registration free for the first year of life, alongside awareness and outreach, is expected to raise registration rates, particularly among disadvantaged populations.

#### 4.5.7. Partnerships With International Organizations

The Ghanaian government has partnered with international organizations such as UNICEF and the UNDP to enhance birth registration infrastructure and personnel training. These partnerships provide both technical and financial support, enabling the government to implement best practices in registration processes [26] Through training programs, local officials and health workers are equipped with the necessary skills to effectively manage birth registration, ensuring that they can assist families in navigating the registration process. This collaboration not only strengthens the capacity of local systems but also fosters a more robust civil registration framework that can adapt to the needs of the population [26].

### 4.6. Challenges in Birth Registration

Despite noteworthy progress, Ghana continues to encounter several challenges in achieving universal birth registration. These challenges include low awareness of birth registration, religious and cultural influences, geographical and logistical barriers, data management and digitalization issues, the effects of colonial legacy, resource constraints, administrative inefficiencies, and a lack of integration with other services.

#### 4.6.1. Low Awareness of Birth Registration

Low awareness regarding the importance of birth registration is a significant barrier in Ghana, particularly among parents living in rural areas. Many families do not recognize birth registration as a critical priority, resulting in delays or outright neglect of the process. This lack of awareness is often compounded by cultural practices where traditional rituals, such as naming ceremonies, take precedence over legal documentation. For many parents, these cultural customs hold greater significance than the legal implications of failing to register a child's birth. As highlighted by AbouZahr et al. [17], this mindset contributes to a cycle of unregistered births, as families may lack information about the benefits of registration, such as access to healthcare, education, and legal rights. Addressing this issue requires comprehensive awareness campaigns that educate communities on the importance of birth registration and its long-term benefits for children and families. By integrating discussions on legal registration into community events and healthcare settings, stakeholders can foster a greater understanding of its significance and encourage timely registration practices.

#### 4.6.2. Religious and Cultural Influences

A crucial barrier to effective birth registration in Ghana is the influence of religious beliefs and cultural practices on the perception of legal registration. In many communities, traditional naming ceremonies hold significant cultural importance and are often prioritized over the official birth registration process. These ceremonies, perceived as vital rites of passage, can delay or overshadow legal documentation, leading families to neglect the necessity of registering their children's births. Consequently, many families do not view birth registration as a pressing issue, viewing it as less important than fulfilling cultural obligations. This creates a societal norm where legal recognition is considered secondary, further perpetuating the cycle of unregistered births. The prevalence of these traditional practices, particularly among certain religious groups, shapes a cultural perspective that diminishes the perceived importance of government initiatives aimed at encouraging birth registration.

Importantly, gendered dimensions of these cultural and religious practices further reinforce disparities in registration. In some communities, the social preference for male children means that boys are more likely to be prioritized for birth registration, while girls' registration is delayed or overlooked [32–34]. Families may choose to perform elaborate naming rituals for boys and ensure their legal documentation, whereas girls are sometimes perceived as less deserving of immediate formal recognition [35, 36]. This gender bias entrenched in cultural and religious traditions not only distorts official records but also limits girls' future access to education, healthcare, and social protection [28, 33]. Addressing this requires targeted strategies that challenge discriminatory norms while respecting cultural practices. Integrating gender-sensitive messaging into religious and cultural platforms, alongside incentives for the registration of girls, can help shift perceptions and ensure that birth registration becomes both culturally acceptable and equitable [37, 38].

#### 4.6.3. Geographical and Logistical Barriers

Geographical and logistical barriers also hinder birth registration efforts. In some communities, registration offices are scarce, requiring parents to travel long distances to complete the process [19]. The cost and inconvenience associated with these trips discourage many families from registering their children's births. The place of residence of families plays a decisive role in birth registration rates. Urban areas typically show higher registration rates compared to rural locations. In urban centers, there is often better access to registration facilities and services, as well as more awareness regarding the importance of birth registration. Conversely, rural areas may suffer from limited access to these services due to geographical barriers, inadequate infrastructure, or lack of information [19].

#### 4.6.4. Data Management and Digitalization

Inefficient data management remains a significant challenge in Ghana's birth registration system, primarily due to the reliance on manual record-keeping and the limitations of existing digital infrastructure. The manual processes often result in delays, inaccuracies, and a lack of real-time data access, which can hinder effective decision-making and resource allocation. As highlighted by UNICEF, these inefficiencies not only complicate the registration process but also affect the reliability of national statistics on birth rates. In response to these challenges, the Ghanaian government has initiated comprehensive efforts to digitalize birth records. These initiatives include the development of electronic registration systems designed to streamline data collection and management, thereby enhancing operational efficiency. The GSS has indicated that the shift to digital platforms is expected to significantly improve processing times and data accuracy, facilitating better tracking of registered births and ultimately supporting the achievement of universal birth registration in the country. This modernization of data management practices is crucial for strengthening the overall civil registration system and ensuring that every child is recognized legally from birth.

#### 4.6.5. Effects of Colonial Legacy

The legacy of colonial rule has left a lasting impact on the birth registration system in Ghana, which influences current perceptions and practices surrounding birth registration. Established during colonial times, the initial registration framework primarily catered to urban populations, inadvertently neglecting the needs of those in rural areas. This historical oversight has cultivated a belief that birth registration is not a vital element of national development, leading to a lack of prioritization by successive governments. The BDR, which was officially launched in 1965, has faced significant challenges in extending its services beyond urban centers. Consequently, this has resulted in considerable registration gaps, particularly in rural communities where awareness about the importance and process of birth registration remains low. This colonial legacy not only hampers efforts to modernize the registration system but also perpetuates inequities in access to essential services for unregistered children. Recognizing and addressing these historical influences is crucial for reforming the birth registration process and ensuring its integration into broader national development strategies.

#### 4.6.6. Resource Constraints

Ghana confronts several socioeconomic challenges that hinder effective birth registration, primarily due to financial constraints and competing developmental priorities. The government grapples with the pressure to allocate limited resources to urgent sectors including healthcare, education, and infrastructure, often at the expense of funding for birth registration initiatives. As a result, the BDR has historically contended with unpredictable and insufficient financial support, which significantly impairs its ability to carry out effective registration campaigns and outreach programs. The inadequate allocation of funds restricts the registry's capacity to employ personnel, conduct community awareness programs, and implement technological advancements necessary for improving the registration process. This lack of resources is further exacerbated in rural areas, where outreach efforts are essential to inform residents about the importance of birth registration. Ultimately, addressing these resource constraints is crucial to enhancing Ghana's birth registration system, ensuring that all children receive the legal recognition they need for accessing essential services and rights.

#### 4.6.7. Administrative Inefficiencies

The administrative framework governing birth registration in Ghana is characterized by several inefficiencies that impede timely processing and accurate documentation. Outdated laws and bureaucratic hurdles contribute significantly to delays in the registration process. Furthermore, the reliance on manual record-keeping, coupled with inadequate digital infrastructure, exacerbates issues of inaccuracy and slow data retrieval. These persistent inefficiencies often discourage parents from undertaking the registration process, leading to a cycle of unregistered births. Many families perceive the process as cumbersome and overly complex, which can result in them prioritizing other immediate needs over legal documentation. Addressing these administrative challenges is crucial for enhancing the birth registration system and ensuring that all children are legally recognized at birth.

#### 4.6.8. Lack of Integration With Other Services

The lack of integration between birth registration and other essential services, particularly within health systems, poses a considerable challenge to achieving comprehensive coverage. Although strides have been made to link birth registration with maternal and child health initiatives, the efforts are still limited in scope and not implemented uniformly across the country. Many health facilities do not systematically provide birth registration services alongside maternal care, resulting in missed opportunities for early registrations during hospital visits. Without a cohesive strategy that emphasizes the importance of birth registration across various sectors, such as education and social services, birth registration remains a largely standalone process. This separation not only reduces the overall efficiency of the registration system but also fails to leverage existing health infrastructure to promote timely registration, thereby perpetuating the problem of unregistered births.

## 5. Discussion

The question of whether birth registration in Ghana is a national priority, or a neglected necessity requires an examination of governmental commitment, policy implementation, and societal attitudes toward birth registration. Although Ghana has made significant strides in improving birth registration systems, persistent challenges highlight a gap between policy intent and actual outcomes. Applying the HRBA, birth registration is not merely a bureaucratic requirement but a fundamental right of every child, as enshrined in Article 7 of the UNCRC [16]. The UNCRC mandates that all children have the right to a legal identity, including a name and nationality. Similarly, the African Charter on the Rights and Welfare of the Child (ACRWC) underscores birth registration as a mechanism to protect children's rights to nationality, inheritance, and access to social services [39]. Despite these commitments, Ghana continues to grapple with barriers that prevent universal birth registration, raising concerns about the country's ability to fully realize children's rights.

From an HRBA perspective, Ghana's policy efforts should be assessed based on the principles of universality, nondiscrimination, and accountability. Ghana has demonstrated commitment through initiatives such as digitalization, health system integration, and mobile registration units [9, 40]. These efforts align with SDG 16.9, which seeks to provide legal identity for all by 2030. However, prioritizing policy does not necessarily equate to effective implementation. The institutional theory [41] suggests that while organizations may adopt formal policies to align with international norms, actual implementation is often constrained by structural limitations, resource availability, and bureaucratic inefficiencies. To achieve meaningful progress, the government must enact these policies effectively by addressing challenges related to inadequate funding, personnel training, and public awareness campaigns.

Ghana's emphasis on digital birth registration has improved data accuracy and reduced record-keeping errors, yet disparities in accessibility persist. UNICEF [25] reports that digitalization has benefited urban populations more than rural communities, where infrastructure and internet connectivity are limited. From an HRBA standpoint, this urban–rural divide raises concerns about equity and inclusiveness. Comparative studies show that in countries like Estonia and Kenya, comprehensive e-governance strategies have successfully extended digital registration services to remote areas through mobile technology [42]. Ghana can learn from these experiences by investing in mobile registration units equipped with offline capabilities to reach underprivileged communities more effectively.

Despite legislative reforms, such as the free registration of births within the first year of life [30], registration rates remain uneven across socioeconomic groups. According to the MICS [23], children from wealthier households are significantly more likely to be registered than those from lower income families. This supports the capability approach [43], which argues that policy effectiveness is contingent on individuals' ability to access resources and exercise their rights. Ghana's model, while commendable, remains fragmented, as parents often have to make a separate effort to visit registration centers. Bridging this gap requires a holistic approach that embeds birth registration within routine maternal and child health services, ensuring that no child is left undocumented due to logistical barriers.

Birth registration is also shaped by cultural and religious practices. In many Ghanaian communities, traditional naming ceremonies take precedence over formal registration [17]. While the Births and Deaths Registration Act allows for names to be added later [14], many parents delay registration indefinitely, prioritizing cultural rituals over legal requirements. Studies in sub-Saharan Africa have documented similar trends, where community traditions influence birth registration timelines, often leading to low compliance rates [5, 8, 13]. Addressing this issue requires culturally sensitive advocacy that aligns legal registration with traditional practices rather than positioning them as conflicting interests. Religious beliefs can further impede birth registration, particularly among groups that perceive formal documentation as unnecessary or intrusive. Studies have shown that some sects resist official registration due to distrust in government systems [44]. Engaging religious leaders as advocates for birth registration can help mitigate these concerns, as demonstrated in Bangladesh's successful partnership with Islamic clerics to promote civil registration [45].

Gender disparities in birth registration are another critical issue. According to the GSS [28], data indicate that male children are more likely to be registered than female children due to sociocultural preferences for male offspring. This bias not only skews official records but also exacerbates gender-based inequalities, limiting girls' access to future opportunities such as education and social protection. Global comparisons reveal that in India, targeted financial incentives for parents of female children have led to significant improvements in female birth registration rates [46]. Ghana can adopt similar gender-sensitive interventions, such as conditional cash transfers or fee waivers for girls' birth registration, to ensure equitable access.

Birth registration is essential for national planning and resource allocation. Without accurate birth records, governments struggle to effectively distribute resources for healthcare, education, and child protection services [1, 2]. The incomplete registration system in Ghana creates blind spots in demographic data, leading to inefficient service delivery. For instance, Ghana's Free Senior High School (SHS) policy relies on national population data for planning purposes [47]. Inaccurate birth registration data can result in either underestimating or overestimating school enrolment figures, affecting the allocation of educational resources. Comparative studies indicate that Brazil and Thailand have successfully linked birth registration with social protection programs, ensuring that unregistered children do not miss out on essential services [48]. Ghana could strengthen its birth registration system by integrating it with existing welfare programs, thereby incentivizing parents to complete the registration process.

## 6. Limitations and Strengths

This study is based on secondary data analysis, which presents both limitations and strengths. A key limitation is that the desk review depends on the quality, accuracy, and completeness of existing data sources. Some official records may underreport challenges, while NGO and media reports may emphasize particular cases without national representativeness. The lack of access to raw administrative data also limits the ability to conduct more detailed statistical analysis. Furthermore, as most documents reviewed were published between 2010 and 2023, earlier historical dynamics may not be fully captured.

Despite these limitations, secondary data analysis has significant strengths. It allows for the synthesis of diverse perspectives, including government, international agencies, and academic sources, providing a holistic understanding of birth registration in Ghana. The use of multiple sources enhances credibility and helps identify patterns and gaps across different reports. Moreover, the inclusion of global best practices enriches the comparative dimension of the study, making the findings relevant not only for Ghana but also for other sub-Saharan African countries facing similar challenges.

## 7. Conclusion

Despite Ghana's policy commitments and ongoing reforms, birth registration remains an unfinished agenda for many marginalized communities due to persistent gaps in implementation, accessibility, and cultural integration. Moving beyond rhetoric to practical, community-centered solutions is essential to ensure that no child is left undocumented. Grounded in the HRBA, birth registration must be recognized not as an administrative formality but as a fundamental right that underpins children's access to education, healthcare, and protection from exploitation. From this perspective, ensuring universal registration is not optional but a legal and moral obligation of the state. The first priority must be to expand mobile registration services with offline capabilities, a task to be spearheaded by the BDR in collaboration with the Ministry of Local Government and Rural Development to overcome geographical and infrastructural barriers in rural and underserved communities. The second priority is to fully integrate birth registration into maternal and child health services, led by the Ministry of Health and the Ghana Health Service, ensuring that every child delivered in a health facility is automatically registered. A third priority lies in addressing low awareness and cultural resistance by partnering with traditional and religious leaders through coordination by the MoGCSP and the National Commission for Civic Education (NCCE), to normalize registration as a community responsibility. In addition, deliberate efforts must be made to address gender disparities by adopting gender-sensitive incentives, such as conditional cash transfers, under the joint responsibility of MoGCSP and the Ministry of Finance, to encourage equitable registration of girls and boys. Finally, linking registration to social protection programs and modernizing the BDR through digital data systems, interagency collaboration, and staff training should be championed by BDR, the NIA, and the Ministry of Communication and Digitalization. In line with the HRBA, these measures affirm the state's duty to respect, protect, and fulfill the rights of every child, ensuring that birth registration becomes a universal, equitable, and enforceable entitlement rather than a privilege.

### 7.1. Implications for Policy and Practice

The implications of improved birth registration in Ghana extend beyond legal recognition; they have profound impacts on governance, social development, and the protection of children's rights. The HRBA frames birth registration as a core entitlement necessary for the realization of all other rights, including access to healthcare, education, and social protection. To respond to the most pressing challenge of limited accessibility, the BDR, with support from local government structures, should prioritize the expansion of mobile registration units and the development of offline digital platforms for remote areas. Equally urgent is the integration of birth registration with maternal and child health services, where the Ministry of Health and Ghana Health Service must develop automatic protocols to register newborns at delivery points. Once access and integration are secured, the next step is to tackle cultural and informational barriers. Here, the MoGCSP, NCCE, and faith-based organizations should lead targeted community education campaigns, ensuring that traditional and religious leaders are active partners in promoting the legal and developmental benefits of timely registration. Gender inequities should be addressed by MoGCSP in partnership with the Ministry of Finance through gender-sensitive incentives, including financial transfers for registering female births. Finally, a long-term priority is the modernization of governance systems, which should be driven by the BDR in collaboration with the NIA and Ministry of Communication and Digitalization to ensure digitized records, effective staff training, accountability, and strong enforcement of existing legislation. In alignment with HRBA, these priorities emphasize participation, accountability, nondiscrimination, and equity as the guiding principles for policy and practice. By centering children's rights within governance and service delivery, Ghana can transform birth registration into a universally accessible right that strengthens both individual well-being and national development.

In brief, policy recommendations emerging from this study emphasize the need for the BDR to adopt a HRBA that ensures participation, accountability, and nondiscrimination in birth registration. Strengthening intersectoral collaboration, expanding digital registration platforms, and improving enforcement of existing legislation will also be essential. Equally, the BDR must sustain community education and outreach to empower parents and communities to dismantle cultural barriers that affect birth registration in the country. Together, these measures can help Ghana move toward universal, equitable, and efficient birth registration.

## Figures and Tables

**Table 1:** Application of HRBA principles to birth registration in Ghana.

**HRBA principle**	**Ghana's current status**	**Gaps identified**
Participation	Civil society groups (e.g., UNICEF Ghana, Plan International) support awareness campaigns; community mobilization exists in some districts.	Limited engagement of rural communities; low involvement of parents in decision-making about registration processes.
Accountability	The Registration of Births and Deaths Act (Act 1027, 2020) mandates universal registration; Births and Deaths Registry produces annual reports.	Weak enforcement mechanisms; poor monitoring of compliance at district levels; inadequate penalties for nonregistration.
Nondiscrimination	Legal framework guarantees registration for all children regardless of status.	Marginalized groups (rural poor, children of migrants, and nomadic groups) face higher exclusion due to distance, poverty, and lack of documentation.
Empowerment	Government and NGOs have piloted digital registration (e.g., m-Birth project) to reduce access barriers.	Parents' knowledge of rights remains low; lack of sustained public education campaigns in remote areas.
Legality	Ghana is a signatory to CRC and African Charter on the Rights and Welfare of the Child; national law aligns with international obligations.	Weak translation of legal commitments into practice; gaps between statutory requirements and service delivery at community level.

**Table 2:** Databases and search strategy.

**Source/database**	**Search terms used**	**Inclusion years**
Google Scholar	“Birth registration Ghana”, “policy”	2010–2023
PubMed	“Civil registration”, “child rights”	2010–2023
JSTOR	“Birth registration”, “Sub-Saharan Africa”,” gender, culture, and birth registration”.	2010–2023
Scopus	“Legal identity”, “birth registration systems”	2010–2023
Government archives (BDR, GSS)	“Birth statistics”, “registration reports”, “gender and birth registration” and” culture and birth registration”.	2010–2023
UNICEF, UNDP, Plan International repositories	“Birth registration Ghana”, “gender and birth registration” and” culture and birth registration”.	2010–2023

## Data Availability

Data sharing is not applicable to this article as no new data were created or analyzed in this study.
